# Bloodstream Infections in Solid Malignancies: Risk Factors, Resistance Patterns, and Clinical Outcomes from a Two-Center Cohort in Heraklion, Crete

**DOI:** 10.3390/life16050720

**Published:** 2026-04-24

**Authors:** Maria Bachlitzanaki, Georgios Aletras, Petros Ioannou, Eirini Bachlitzanaki, Georgios Hamilos, Georgios Samonis, Diamantis Kofteridis

**Affiliations:** 1 Department of Internal Medicine, Venizelio General Hospital of Heraklion, 71409 Heraklion, Greece; medp2011922@med.uoc.gr; 2 School of Medicine, University of Crete, 71003 Heraklion, Greecehamilos@uoc.gr (G.H.); samonis@med.uoc.gr (G.S.); 3 Department of Cardiology, Venizelio General Hospital of Heraklion, 71409 Heraklion, Greece; medp2012222@med.uoc.gr (G.A.); eirinibach@outlook.com.gr (E.B.); 4 Department of Internal Medicine and Infectious Diseases, University Hospital of Heraklion, 71003 Heraklion, Greece; 5 Department of Clinical Microbiology and Microbial Pathogenesis, School of Medicine, University of Crete, 71003 Heraklion, Greece; 6 First Oncology Department, Metropolitan Hospital, Neon Faliron, 18547 Athens, Greece

**Keywords:** bloodstream infection, solid tumors, antimicrobial resistance, polymicrobial infection, multidrug-resistant pathogens, infection-related mortality

## Abstract

**Background**: Bloodstream infections (BSIs) represent a severe complication among patients with solid tumors (STs), contributing substantially to morbidity and mortality, especially in the context of increasing antimicrobial resistance (AMR). Evidence in STs remains limited compared to hematologic malignancies. **Methods**: We conducted a two-center observational study including 282 hospitalized adults with solid malignancies and documented infections; 66 patients (23.4%) had confirmed BSIs. Clinical characteristics, microbiological profiles, resistance patterns, and outcomes were evaluated. **Results**: Patients had a mean age of 66.4 ± 11.8 years, and 63.6% were male. The majority (84.8%) had stage IV disease and were receiving chemotherapy (95.5%). Gram-negative bacteria predominated (51.5%), mainly *Escherichia coli*, *Klebsiella pneumoniae*, and *Pseudomonas aeruginosa*. Multidrug resistance (MDR) was detected in 45.5% of isolates, while 28.8% of BSIs were polymicrobial; both were associated with worse outcomes and increased infection-related mortality (*p* = 0.041 and *p* = 0.032, respectively). Overall infection-related mortality reached 28.8%. In multivariable analysis, poor functional status (ECOG PS ≥ 2), prior hospitalization, and concurrent radiotherapy were independently associated with unfavorable outcomes. **Conclusions**: BSIs remain a significant cause of morbidity and mortality in patients with STs. The high burden of Gram-negative and MDR pathogens, along with the impact of polymicrobial infections and impaired performance status, highlights the need for early risk stratification and locally adapted empirical antimicrobial strategies. Prospective multicenter studies are warranted to validate these findings and optimize management strategies in this vulnerable population.

## 1. Introduction

Bloodstream infections (BSIs) represent one of the most severe infectious complications in oncology and remain a major cause of morbidity and mortality among cancer patients [1]. Although BSIs in hematologic malignancies have been extensively described, data in patients with solid tumors (STs) are limited [2,3,4].

Patients with STs are at increased risk of developing a BSI due to frequent use of invasive devices as well as to immunosuppression from chemotherapy, corticosteroids, or neutropenia, and disruption of mucosal barriers. These factors facilitate translocation of organisms from endogenous flora or hospital environments into the bloodstream [4].

Furthermore, the rising global burden of antimicrobial resistance (AMR) further complicates the management of BSIs in cancer patients [5]. The increasing prevalence of multidrug-resistant (MDR) poses significant challenges to empirical antimicrobial treatment, often leading to delays in effective therapy and poorer clinical outcomes [6,7,8]. With regard to the microbiological epidemiology in patients with STs, recent trends suggest a prevalence of Gram-positive organisms linked to increased use of devices as well as increased hospital exposure [9,10].

Moreover, polymicrobial BSIs, although less frequently reported, may be associated with adverse outcomes including increased risk of septic shock and mortality compared to monomicrobial bacteremia. Gram-negative organisms predominate, but *Enterococcus* spp., *Candida* spp., and multidrug-resistant strains are more frequently isolated in polymicrobial episodes [4,11].

Identifying clinical predictors of adverse outcomes in BSIs, and thereby enabling early recognition of high-risk patients, may improve timely diagnostic evaluation, optimize empirical antimicrobial therapy, and facilitate appropriate escalation of care. Performance status, disease stage, prior healthcare exposure, and treatment-related adverse events such as mucositis or neutropenia have been proposed as potential prognostic factors; however, the majority of available data derive from studies in hematologic malignancies while data focusing on patients with solid tumors remain limited [11].

As a result, existing risk stratification tools may not demonstrate the same predictive performance in the more diverse group of patients with solid tumors [3,9,12]. Moreover, relatively few studies have simultaneously evaluated clinical predictors, microbiological characteristics, and resistance profiles in real-world cohorts of BSIs in this population [1,6,10,12].

Consequently, the need to better characterize BSIs in patients with neoplasia, regarding clinical, microbiological, and resistance-related parameters, remains crucial to guide empirical antimicrobial treatment and improve outcomes [4,6,9,12].

The aim of this observational two-center study was to describe the clinical characteristics, microbiological spectrum, antimicrobial resistance patterns, and outcomes of BSIs in adult patients with solid tumors.

## 2. Materials and Methods

### 2.1. Patient Sample

A prospective observational study encompassing all BSIs in 66 patients with STs was conducted. These patients were receiving intravenous or oral (per os) chemotherapy or immunotherapy while hospitalized in the oncology departments of the two largest tertiary hospitals on the island of Crete, Greece—the University General Hospital of Heraklion and the Venizelio General Hospital of Heraklion—during the study period from January 2020 to December 2021.

Clinical and epidemiological characteristics of the patients were recorded, including demographic characteristics (age, sex), comorbidities, cancer type and stage, performance status, and details of ongoing anticancer therapy (type and duration). Additional variables included the presence of indwelling devices, with documentation of placement and removal dates, prior hospitalizations or invasive procedures, recent antimicrobial or corticosteroid exposure, cause and duration of hospitalization, fever characteristics, type and duration of antimicrobial treatment (including modifications and reasons for change), and clinical outcomes of infection and overall hospitalization. The presence of mucositis—and the use of hematopoietic growth factors—specifically granulocyte colony-stimulating factors (G-CSF)—were also documented. Microbiological cultures were obtained from blood, urine, sputum, catheter tips, pus, or stool, and potential pathogens were identified using standard laboratory microbiological methods. Microorganism identification and antimicrobial susceptibility testing were performed using standard microbiological laboratory methods routinely applied in the participating centers. Antimicrobial susceptibility results were interpreted according to the European Committee on Antimicrobial Susceptibility Testing (EUCAST) criteria. Resistance mechanisms, including methicillin resistance and extended-spectrum β-lactamase (ESBL) production, were identified based on routine phenotypic methods and laboratory reporting in the participating centers. The causative organisms and their antimicrobial resistance profiles were recorded. Multidrug-resistant (MDR), extensively drug-resistant (XDR) and pandrug-resistant (PDR) organisms were defined according to internationally accepted criteria (). MDR was defined as non-susceptibility to at least one agent in three or more antimicrobial categories and, while XDR was defined as non-susceptibility to at least one agent in all but two or fewer antimicrobial categories and PDR as non-susceptibility to all agents in all antimicrobial categories [13]. Each bloodstream infection episode was analyzed at the patient level. In polymicrobial infections, all clinically relevant isolates were included in the analysis.

A polymicrobial infection is defined as an infection caused by two or more microorganisms, including bacteria, viruses, fungi, or parasites. Such infections commonly arise in settings where multiple pathogens coexist, including surgical wounds, abscesses, or intra-abdominal infections. Interactions among co-infecting pathogens may enhance virulence, promote antimicrobial resistance, and increase disease severity [14].

Central line–associated bloodstream infection (CLABSI) was defined according to Centers for Disease Control and Prevention (CDC) surveillance criteria as the isolation of a recognized pathogen from at least one blood culture (or, in the case of common skin commensals, from at least two separate blood cultures drawn on separate occasions) in a patient with a central venous catheter in place at the time of infection or within 48 h prior to onset, in the absence of an alternative source of infection and not present at admission [15].

The dominant pathogen refers to the microorganism that plays the most significant role in driving the infectious process, although in some cases the clinical phenotype may result from the combined effect of multiple pathogens. It is typically identified as the organism associated with the highest microbial burden, greater virulence, and increased risk of complications or adverse outcomes. Clinical improvement is usually observed when targeted therapy is directed against this organism, even when other microorganisms are present [16].

The study enrolled patients with active solid malignancies undergoing intravenous or per os chemotherapy or immunotherapy who were diagnosed with microbiologically documented BSI, either at hospital admission or during hospitalization.

Patients with hematologic malignancies, those undergoing hematopoietic stem cell transplantation and those who did not meet the diagnostic criteria for BSI, as defined above, were excluded from the study.

### 2.2. Statistical Analysis

Statistical analyses were performed using IBM SPSS Statistics version 29.0 (IBM Corp., Armonk, NY, USA). Continuous variables were tested for normality using the Kolmogorov–Smirnov test and visual inspection of P–P plots. Normally distributed variables were expressed as mean ± standard deviation, while non-normally distributed variables were presented as median and interquartile range (IQR). Categorical variables were expressed as frequencies and percentages.

Comparisons between quantitative variables were performed using Student’s *t*-test or one-way analysis of variance (ANOVA) for normally distributed data, and the Mann–Whitney U or Kruskal–Wallis tests for non-parametric data. Associations between categorical variables were assessed using the Chi-square test. Multivariate logistic regression analysis was applied to identify independent predictors of infection-related mortality and other outcomes. A two-sided *p*-value ≤ 0.05 was considered statistically significant.

### 2.3. Study Approval

This study was approved by the scientific committee of the University General Hospital of Heraklion, Crete (Protocol number: 74/3/11-3-2020), the scientific committee of the Venizelio General Hospital of Heraklion, Crete (Protocol number: 4/1/29-01-2020), and the Ethics committee of the University of Crete (Protocol number: 9644/15-1-2020). The specifications and objectives of the study followed the ethical guidelines of the Declaration of Helsinki (1975) and complied with the General Data Protection Regulation (GDPR).

## 3. Results

Among the 282 hospitalized patients with STs included in the study, 66 patients (23.4%) with confirmed BSIs were included in the final analysis. The mean age was 66.4 ± 11.8 years, and 42 (63.6%) were male. Gastrointestinal malignancies constituted the most common primary cancer type, with colorectal cancer (CRC) representing 19.7%, followed by pancreatic cancer (13.6%) and non–small cell lung cancer (12.1%). The vast majority of patients had advanced disease, with 84.8% diagnosed at stage IV, and 56.1% exhibiting progressive disease at the time of infection. Baseline patient characteristics, clinical risk factors, and their association with outcomes are summarized in Table 1.

Regarding functional status, the majority of patients had a favorable Eastern Cooperative Oncology Group (ECOG) performance status, with 63.6% classified as ECOG PS 0–1, compared with 36.4% of patients presenting with impaired performance status (ECOG PS ≥ 2) (Appendix A).

Common comorbidities included hypertension (39.4%), diabetes mellitus (34.8%), smoking history (22.7%), dyslipidemia (18.2%), and vascular disease (15.2%).

Exposure to healthcare and treatment-related risk factors was frequent. Previous hospitalization within the last 3 months was documented in 25 of 66 (37.9%), while 30 patients (45.5%) had received antibiotics—reflecting both therapeutic and empirical use without specific distinction of prophylactic administration—and 6 patients (9.1%) had received antifungals in the preceding 3 months. Mucositis was reported in 7 patients (10.6%), and 11 patients (16.7%) were neutropenic either at presentation or during the course of infection. Most patients (43/66; 65.2%) had been treated with G-CSF prior to admission.

Chemotherapy was the predominant treatment modality, administered to 95.5% of patients, whereas 4.5% received immunotherapy, and 6.1% underwent concurrent radiotherapy. Central venous catheters (CVCs) were present in 65.2% of cases, and 24.2% of patients carried at least one additional indwelling medical device.

Among the 66 patients, 26 (39.4%) were diagnosed with central line–associated bloodstream infections (CLABSIs), while 10 patients (16.7%) presented with febrile neutropenia (FN). Primary BSIs accounted for the majority of cases (56/66; 84.8%), whereas secondary BSIs represented 10 of 66 patients (15.2%), with no significant difference in clinical outcomes between the two categories.

The vast majority of patients (95.5%) developed fever during the infectious episode, and 12.1% experienced recurrent febrile episodes. Fever recurrence was strongly associated with increased infection-related mortality (62.5% vs. 13.8%, *p* = 0.011).

The median duration of hospitalization was 12 days (IQR 7–18). Empirical antimicrobial therapy of median duration of 14.5 days (IQR 10–18) was administered in all patients. Modification or escalation of antimicrobial therapy occurred in 34.8% of cases, either due to microbiological culture results (34.8%), clinical deterioration (30.4%), or recurrence of fever (34.8%). Antifungal therapy was administered in 10.6% of patients for a median of 8 days (IQR 2–15), while antiviral treatment was required in 1 patient (1.5%).

Central venous catheters were present in 43 of 66 patients (65.2%), whereas 16 patients (24.2%) carried at least one additional indwelling medical device. Neither the presence of a CVC nor other foreign bodies was significantly associated with infection outcome (*p* = 0.720 and *p* = 0.459, respectively).

Bacterial pathogens predominated, while fungal isolates were detected in 3 patients (4.5%), including two cases of *Candida albicans* and one case of *Candida tropicalis*. Gram-negative bacteria slightly predominated over Gram-positive organisms (51.5% vs. 48.5%, respectively). *Escherichia coli*, *Klebsiella pneumoniae*, and *Pseudomonas aeruginosa* were the leading *Gram-negative bacteria* and *Staphylococcus hominis*, *Staphylococcus epidermidis*, and *Staphylococcus aureus* were the most common Gram-positive pathogens.

Polymicrobial BSIs were identified in 19 patients (28.8%). To further explore their clinical impact, a subgroup analysis was performed. Polymicrobial episodes were associated with significantly higher infection-related mortality compared with monomicrobial infections (31.6% vs. 14.9%, *p* = 0.032).

Antimicrobial susceptibility testing was performed for all isolates, among which 45.5% demonstrated antimicrobial resistance, encompassing different resistance phenotypes. Methicillin-resistant staphylococci accounted for 63.3% of resistant isolates, while 20% fulfilled criteria for multidrug resistance (MDR), of which 66.7% were extended-spectrum β-lactamase (ESBL)—producing organisms. The distribution of causative pathogens and their antimicrobial resistance profiles is summarized in Table 2 and Table 3 respectively.

Resistance profile was significantly associated with patient outcome (*p* = 0.041), with those infected by susceptible pathogens showing higher improvement rates (77.8%). Resistance type also influenced cause of death (*p* < 0.001), with methicillin resistance being strongly correlated with infection-related mortality. Although these findings were significant, they should be interpreted cautiously due to the limited sample size.

Analysis of clinical and disease-related variables identified several factors significantly associated with adverse outcomes and infection-related mortality. Male sex was associated with infection-related mortality in univariate analysis (*p* = 0.025), although this finding was not a primary focus of the study. Functional status also emerged as one of the most significant prognostic indicators. Overall, 24 of 66 patients (36.4%) presented with impaired performance status (ECOG PS ≥ 2), and this functional impairment was strongly associated with both overall outcome (*p* = 0.009) and infection-related mortality (*p* = 0.009). Patients with preserved performance status (ECOG PS 0–1) exhibited a markedly higher likelihood of clinical improvement compared with those with impaired functional status.

Prior hospitalization within the previous three months, reported in 25 patients (37.9%), was another adverse prognostic factor, significantly associated with patient outcome (*p* = 0.039).

As previously described, recurrence of fever, observed in 12.1% of patients, was significantly linked to increased infection-related mortality (62.5% vs. 13.8%, *p* = 0.011), suggesting its potential role as an early clinical indicator of poor prognosis. In contrast, previous exposure to antibiotics or antifungal agents, recent surgical or invasive procedures, and the presence of central venous catheters or other indwelling devices were not significantly correlated with clinical outcome.

Regarding cancer therapy, radiotherapy administered during the infectious episode was associated with markedly higher mortality (100% vs. 14.5%, *p* < 0.001) and demonstrated a strong association with infection as the primary cause of death (*p* = 0.003). Disease response at the time of infection was also significantly correlated with prognosis (*p* = 0.019), with progressive disease being linked to worse outcomes.

Finally, polymicrobial BSIs (28.8% of cases) and specific antimicrobial resistance patterns were correlated with significantly higher infection-related mortality (*p* < 0.001).

## 4. Discussion

The present study identified impaired performance status, prior hospitalization, recurrent fever, concurrent radiotherapy, progressive disease, polymicrobial infection, and antimicrobial resistance as key determinants of adverse outcomes in patients with STs and BSIs, underscoring the importance of early risk recognition and tailored management strategies.

Solid tumors represent a highly heterogeneous group of malignancies, and bloodstream infections (BSIs) remain a serious and potentially life-threatening complication despite major advances in oncologic therapies [4]. BSIs result from the translocation of pathogenic microorganisms into the bloodstream and constitute one of the leading causes of infection-related mortality in oncology patients, with an estimated incidence of 5.5 per 1000 hospitalizations [2]. Infections in patients with STs are increasingly recognized as a clinically relevant concern. Although the relative frequency of BSIs is lower in patients with STs, the overall burden of this patient population is substantially higher, making BSIs an important issue in everyday clinical practice [17]. In the present two-center cohort, 66 of 282 patients (23.4%) had a microbiologically documented BSI, including febrile neutropenia-associated and secondary infections—a proportion that appears higher than that reported in several previous cohorts [9].

Several risk factors for BSIs have been identified, with the presence of foreign bodies, including CVCs and other indwelling devices as well as neutropenia being the most important [1,3,18]. The high prevalence of CVC use in our cohort aligns with previous reports describing their widespread utilization among patients with solid tumors [10]. However, in our study, neither the presence of CVCs nor other indwelling devices was significantly associated with infection-related outcomes, although the limited sample size precludes firm conclusions regarding their prognostic impact.

In the present study, 29% of the patients with STs developed a polymicrobial BSI, which was associated with higher mortality compared with monomicrobial episodes. Although the available literature regarding such polymicrobial BSIs is very limited, existing studies report a variable prevalence of polymicrobial episodes in oncology populations (often ranging broadly between approximately 8–30% depending on cohort characteristics), with several cohorts demonstrating associations with adverse clinical outcomes compared to monomicrobial bacteremia [11,12].

Polymicrobial bloodstream infections represent a complex clinical entity and were identified in a substantial proportion of our cohort. These infections often involve interactions between multiple pathogens, which may enhance virulence, promote antimicrobial resistance, and complicate treatment strategies. In our study, polymicrobial episodes were primarily composed of bacterial pathogens, occasionally accompanied by fungal isolates, reflecting the typical microbiological profile of bloodstream infections in patients with solid tumors. Although polymicrobial infections may also include viruses or parasites, such pathogens were not identified in our cohort. Importantly, polymicrobial infections were associated with worse clinical outcomes, supporting their role as a marker of increased clinical severity and host vulnerability.

Compared to previous studies, the prevalence of polymicrobial BSIs observed in our cohort was at the upper end of the reported spectrum. In a large prospective study, Royo-Cebrecos et al. reported polymicrobial BSIs in approximately 10.2% of cancer-related bacteremia episodes, with significantly higher early and overall case-fatality rates compared with monomicrobial infections [11].

The markedly higher prevalence of polymicrobial BSIs in our study (29%) likely reflects differences in patient characteristics and clinical context, including advanced-stage disease, frequent prior healthcare exposure, and extensive use of invasive devices. In addition, temporal changes in oncologic care, increasing antimicrobial pressure, and rising antimicrobial resistance may predispose contemporary cancer populations to more complex infectious phenotypes.

As documented by other investigators, Gram-negative organisms and *Enterococcus* spp. predominated in polymicrobial episodes and were frequently associated with multidrug-resistant phenotypes—findings that closely mirror the microbiological patterns observed in our cohort [3,12].

Taken together, these findings support the concept that polymicrobial BSIs represent a marker of advanced clinical vulnerability rather than merely a microbiological finding, warranting early risk stratification, heightened clinical vigilance, and prompt initiation of appropriate empiric antimicrobial therapy.

In contrast, earlier cohorts reported by Gudiol et al. and Amanati et al. described lower frequencies of polymicrobial BSIs, likely reflecting differences in patient selection, antimicrobial exposure, and intensity of oncologic treatment [1,9].

Gastrointestinal malignancies were the predominant primary cancer types in our cohort, with colorectal cancer accounting for 19.7% of BSI cases, followed by pancreatic (13.6%) and non-small-cell lung cancer (12.1%). The predominance of gastrointestinal malignancies in our cohort may partly explain the observed distribution of Gram-negative pathogens. The increased susceptibility of colorectal cancer patients to BSIs may be attributed to disruption of the intestinal barrier, facilitating bacterial translocation into the bloodstream. Mechanisms contributing to this include direct tumor invasion, ulceration, mucosal microleakage, and microbiota alterations favoring enteric pathogens such as *Escherichia coli*, *Klebsiella pneumoniae*, and *Enterococcus faecalis* [19,20]. Surgical interventions, postoperative complications, neutropenia, and mucositis induced by chemotherapy further increase the risk of bacteremia. Therefore, early recognition and prompt initiation of appropriate antimicrobial therapy remain essential in this subgroup [21,22].

Beyond immunosuppression, both chemotherapy and radiotherapy contribute to intestinal barrier injury, increasing mucosal permeability, altering microbiota composition, and impairing local immune defenses [23,24]. Islas-Muñoz et al. [10] and Gudiol et al. [9] have demonstrated that abdominal or pelvic radiotherapy increases the risk of enteric BSIs, particularly from *E. coli*, *Enterococcus* spp., and *Clostridioides difficile*. In our study, 95.5% of patients were receiving chemotherapy and 6.1% concurrent radiotherapy. Although the number of patients receiving radiotherapy was small, radiotherapy during the infectious episode was strongly associated with infection-related mortality, resulting in markedly higher infection-related mortality (100% vs. 14.5%, *p* < 0.001). Given the limited sample size, this finding should be interpreted cautiously and considered hypothesis-generating, requiring confirmation in larger cohorts.

Our microbiological findings confirm that patients with solid tumors remain at high risk for BSIs, with Gram-negative pathogens—primarily *K. pneumoniae*, *E. coli*, and *P. aeruginosa*—slightly predominating over Gram-positive organisms (51.5% vs. 48.5%). These observations are consistent with previous reports by Ong et al. [3] and Xue et al. [12], who also described Gram-negative predominance in oncology-associated BSIs, though with greater disparity between Gram populations. Other studies similarly highlight the increasing burden of Gram-negative infections in this setting [1].

A high prevalence of antimicrobial resistance was observed, with 45.5% of isolates exhibiting resistance, including methicillin-resistant staphylococci and multidrug-resistant (MDR) pathogens. These results are comparable to findings from Amanati et al. [1] and Li et al. [25], who reported high prevalence of MDR Gram-negative bacteria and the association of MRSA with increased mortality. In our cohort, resistance phenotypes were significantly associated with both clinical outcome (*p* = 0.041) and cause of death (*p* < 0.001), highlighting their prognostic relevance and the need for locally adapted empirical antimicrobial strategies. Nevertheless, larger multicenter cohorts are required to further validate these associations and explore pathogen-specific mortality patterns.

Fungal BSIs, although infrequent, represent an emerging challenge in immunocompromised cancer patients [26]. *Candida* species and among them *Candida albicans* are the most frequent fungal isolates although a shift to non-*albicans* species has been reported [26]. Fungal pathogens account for approximately 3.1% of BSIs, ranked as the ninth most frequent pathogens causing BSI while the overall mortality of fungal BSIs approaches 50%, and lack of antifungal therapy is a recognized independent risk factor for death [26]. Known predisposing factors include the presence of central lines, invasive procedures, parenteral nutrition, immunosuppressive therapy, and colonization of *Candida* spp., all of which are prevalent in patients with malignancies [27]. In our cohort, three patients (4.5%) developed mixed bacterial-fungal BSIs involving *Candida albicans* or *C. tropicalis*; all three received targeted therapy but ultimately succumbed, illustrating the poor prognosis of such infections. These findings echo those of Vásquez-Olvera et al., who documented *C. albicans* as the most frequent species in oncologic patients, followed by *C. glabrata* and *C. tropicalis* [28].

The median duration of hospitalization in our study was 12 days (IQR 7-18), shorter than that reported by Xue et al. of mean 24.5 days [12]. Infection-related mortality (28.8%) was comparable to previous studies, where mortality ranged between 20–30% depending on pathogen and resistance profile [9,25]. BSIs occurring by certain pathogens could increase mortality rates in patients with solid tumors as reported by the study of Zhang et al. where *E. coli* BSIs showed an infection-related mortality of 35% [29], though notably higher than 10.5% of our cohort, suggesting potential benefits from early empirical coverage and multidisciplinary management.

Our findings are consistent with existing literature indicating that functional status, prior healthcare exposure, antimicrobial resistance, and concurrent radiotherapy are major determinants of prognosis in patients with solid tumors and BSIs. Incorporating these variables into clinical decision-making could enhance early risk stratification, optimize empirical antimicrobial selection, and guide infection control strategies in oncology practice. These findings may help inform locally adapted empirical antimicrobial algorithms in oncology wards with high MDR prevalence.

In conclusion, BSIs represent a common and clinically significant complication among hospitalized patients with solid tumors, contributing substantially to morbidity and mortality. In this real-world, two-center cohort, Gram-negative bacteria were predominant, and nearly half of the isolates demonstrated multidrug resistance. Polymicrobial bloodstream infections were associated with worse clinical outcomes and higher infection-related mortality, underscoring the importance of early recognition and targeted therapeutic strategies.

Impaired functional status (ECOG PS ≥ 2), prior hospitalization, and concurrent radiotherapy emerged as key determinants of prognosis, highlighting the need for individualized risk assessment and prompt empirical coverage tailored to local resistance patterns.

This study is subject to certain limitations, including the involvement of only two medical centers and a relatively small cohort. The vast majority of patients were treated with chemotherapy while only a small proportion were treated with immunotherapy, precluding meaningful subgroup comparisons. The heterogeneity of the study population may have influenced outcome distributions and limited causal inference despite multivariate adjustments. In addition, routine surveillance cultures for colonization (e.g., rectal or throat swabs) were not systematically performed, as this was not part of standard clinical practice in the participating centers. As a result, the potential impact of baseline colonization on subsequent bloodstream infections and antimicrobial resistance patterns could not be assessed. Detailed source attribution of bloodstream infections (e.g., environmental or ward-specific origin) was not systematically evaluated, which limits further interpretation of pathogen transmission patterns. Incidence density per bed-days was not calculated, as ward-level denominator data were not available. Although prior antibiotic use was recorded, this information did not distinguish between therapeutic and prophylactic administration. Therefore, the potential impact of antibiotic prophylaxis on bloodstream infections and antimicrobial resistance patterns could not be specifically assessed. Furthermore, although data regarding the time interval from cancer diagnosis to bloodstream infection were available, this variable was not included in the analysis, as it was beyond the predefined scope of the study. Additionally, the timing of infection in relation to neutropenic phases was not systematically recorded. Finally, part of the study period coincided with the COVID-19 pandemic, which likely affected hospitalization patterns and case recording, particularly among infected oncology patients managed in dedicated isolation units, potentially affecting the observed outcomes. Despite these limitations, the study provides valuable real-world data on bloodstream infections in patients with solid tumors, integrating clinical, microbiological, and antimicrobial resistance parameters in a contemporary cohort.

Overall, our findings emphasize the importance of continuous surveillance of infection epidemiology in oncology settings and reinforce the critical role of antimicrobial stewardship, infection control, and multidisciplinary collaboration in optimizing outcomes for cancer patients. Future multicenter studies involving larger and more homogeneous cohorts are warranted to validate these observations and further refine clinical decision pathways for managing BSIs in solid tumor patients.

## Figures and Tables

**Table 1:** Patients’ characteristics and risk factors in correlation with outcomes in patients with BSIs.

Patient Characteristics	All Patients *n* (%)	Outcome *n* (%)				*p*-Value
		Cure	Improvement	Failure	Death	
	*n* = 66	*n* = 5	*n* = 41	*n* = 1	*n* = 19	
**Age**	66.4 ± 11.8 years					
**Sex** **Male** **Female**	42 (63.6) 24 (36.4)	3 (60.0) 2 (40.0)	25 (61.0) 16 (39.0)	1 (100.0) 0 (0.0)	13 (68.4) 6 (31.6)	0.025
**PS (ECOG)** **0** **1** **2** **3** **4**	21 (31.8) 21 (31.8) 11 (16.7) 9 (13.6) 4 (6.1)	2 (40.0) 2 (40.0) 1 (20.0) 0 (0.0) 0 (0.0)	15 (36.6) 14 (34.1) 6 (14.6) 5 (12.2) 1 (2.4)	1 (100.0) 0 (0.0) 0 (0.0) 0 (0.0) 0 (0.0)	3 (15.8) 5 (26.3) 4 (21.1) 4 (21.1) 3 (15.8)	0.009
**Malignancy** **NSCLCSCLC** **Breast** **Esophageal** **Gastric** **CRC** **Pancreatic** **Hepatobiliary** **Bladder** **Prostate** **Uterine** **Endometrial** **Ovarian** **Head and Neck** **CNS** **Sarcoma** **Occult primary**	8 (12.1) 5 (7.6) 3 (4.5) 2 (3.0) 2 (3.0) 13 (19.7) 9 (13.6) 2 (3.0) 2 (3.0) 5 (7.6) 4 (6.0) 1 (1.5) 2 (3.0) 2 (3.0) 2 (3.0) 1 (1.5) 3 (4.5)	1 (20.0) 1 (20.0) 0 (0.0) 0 (0.0) 1 (20.0) 0 (0.0) 0 (0.0) 0 (0.0) 0 (0.0) 1 (20.0) 0 (0.0) 0 (0.0) 0 (0.0) 0 (0.0) 0 (0.0) 0 (0.0) 1 (20.0)	6 (14.6) 2 (4.9) 2 (4.9) 1 (2.4) 0 (0.0) 10 (24.4) 7 (17.1) 1 (2.4) 1 (2.4) 4 (9.8) 2 (4.9) 1 (2.4) 0 (0.0) 1 (2.4) 1 (2.4) 1 (2.4) 1 (2.4)	0 (0.0) 0 (0.0) 0 (0.0) 0 (0.0) 0 (0.0) 1 (100.0) 0 (0.0) 0 (0.0) 0 (0.0) 0 (0.0) 0 (0.0) 0 (0.0) 0 (0.0) 0 (0.0) 0 (0.0) 0 (0.0) 0 (0.0)	1 (5.3) 2 (10.5) 1 (5.3) 1 (5.3) 1 (5.3) 2 (10.5) 2 (10.5) 1 (5.3) 1 (5.3) 0 (0.0) 2 (10.5) 0 (0.0) 2 (10.5) 1 (5.3) 1 (5.3) 0 (0.0) 1 (5.3)	0.967
**Stage** **0** **I** **II** **III** **IV**	0 (0.0) 1 (1.5) 4 (6.1) 5 (7.6) 56 (84.8)	0 (0.0) 0 (0.0) 1 (20.0) 0 (0.0) 4 (80.0)	0 (0.0) 0 (0.0) 3 (7.3) 2 (4.9) 36 (87.8)	0 (0.0) 0 (0.0) 0 (0.0) 0 (0.0) 1 (100.0)	0 (0.0) 1 (5.3) 0 (0.0) 3 (15.8) 15 (78.9)	0.524
**Disease Response** **SD** **PD** **PR** **CR**	28 (42.4) 37 (56.1) 1 (1.5) 0 (0.0)	3 (60.0) 2 (40.0) 0 (0.0) 0 (0.0)	21 (51.2) 19 (46.3) 1 (2.4) 0 (0.0)	1 (100.0) 0 (0.0) 0 (0.0) 0 (0.0)	3 (15.8) 16 (84.2) 0 (0.0) 0 (0.0)	0.019
**Prior Hospitalization** **No** **Yes**	41 (62.1) 25 (37.9)	4 (80.0) 1 (20.0)	28 (68.3) 13 (31.7)	1 (100.0) 0 (0.0)	8 (42.1) 11 (57.9)	0.039
**Oropharyngeal mucositis** **No** **Yes**	59 (89.4) 7 (10.6)	4 (80.0) 1 (20.0)	39 (95.1) 2 (4.9)	1 (100.0) 0 (0.0)	15 (78.9) 4 (21.1)	0.173
**Prior antimicrobial use** ** (last 3 months)** **No** **Yes**	36 (54.5) 30 (45.5)	3 (60.0) 2 (40.0)	23 (56.1) 18 (43.9)	1 (100.0) 0 (0.0)	9 (47.4) 10 (52.6)	0.528
**Prior antifungal use** ** (last 3 months)** **No** **Yes**	60 (90.9) 6 (9.1)	5 (100.0) 0 (0.0)	38 (92.7) 3 (7.3)	1 (100.0) 0 (0.0)	16 (84.2) 3 (15.8)	0.467
**G-CSF** **No** **Yes**	23 (34.8) 43 (65.2)	1 (20.0) 4 (80.0)	15 (36.6) 26 (63.4)	1 (100.0) 0 (0.0)	6 (31.6) 13 (68.4)	0.475
**Cancer therapy** **Chemotherapy** **Immunotherapy**	63 (95.5) 3 (4.5)	4 (80.0) 1 (20.0)	39 (95.1) 2 (4.9)	1 (100.0) 0 (0.0)	19 (100.0) 0 (0.0)	0.131
**Current radiotherapy** ** (last 1 month)** **Yes** **No**	4 (6.1) 62 (93.9)	0 (0.0) 5 (100.0)	0 (0.0) 41 (100.0)	0 (0.0) 1 (100.0)	4 (21.1) 15 (78.9)	0.002

**PS:** Performance status, **ECOG:** Eastern Cooperative Oncology Group, **NSCLC:** Non-small cell lung cancer, **SCLC:** Small cell lung cancer, **CRC:** Colorectal cancer, **SD:** Stable disease, **PD:** Progressive disease, **PR:** Partial response, **CR:** Complete response, **G-CSF:** Granulocyte colony-stimulating factor.

**Table 2:** Distribution of isolated pathogens in patients with BSI.

Isolated Pathogens					
Gram+ (*n* = 32)			Gram- (*n* = 34)		
	*n*	%		*n*	%
*Staphylococci* *S. aureus* *CoNS*	29 4 25	90.6 12.5 78.1	*Klebsiella* spp. *Kl. pneumoniae* *Kl. oxytoca*	8 7 1	23.5 20.6 2.9
*E. faecium*	1	3.1	*Escherichia coli*	13	38.2
*Viridans group streptococci*	1	3.1	*Pseudomonas aeruginosa*	5	14.7
*Micrococcus luteus*	1	3.1	*Enterobacter* spp. *E. cloacae* *E. aerogenes*	3 2 1	8.8 5.9 2.9
			*Salmonella* spp.	2	5.9
			*Bacteroides fragilis*	1	2.9
			*Haemophilus influenzae*	1	2.9
			*Stenotrophomonas maltophilia*	1	2.9

**CoNS:** coagulase-negative staphylococci.

**Table 3:** Antimicrobial resistance patterns among isolates with detected resistance (*n* = 30).

Antimicrobial Resistance		
(*n* = 30)		
	*n*	%
**Methicillin resistant staphylococci**	19	63.3
**MDR Gram-positive**	4	13.3
**MDR Gram-negative**	6	20.0
**XDR Gram-positive**	0	0.0
**XDR Gram-negative**	1	3.3

Antimicrobial susceptibility testing was performed for all isolates; resistance was identified in 30 isolates and is presented accordingly. **MDR:** multidrug-resistant, **XDR:** extensively drug-resistant.

## Data Availability

The data presented in this study are available on request from the corresponding authors.

## Abbreviations

The following abbreviations are used in this manuscript:

AMR	Antimicrobial resistance
ANOVA	Analysis of variance
BSI	Bloodstream infection
CDC	Centers for Disease Control and Prevention
CLABSI	Central line–associated bloodstream infection
CoNS	Coagulase-negative staphylococci
CR	Complete response
CRC	Colorectal cancer
CVC	Central venous catheter
ECOG	Eastern Cooperative Oncology Group
ESBL	Extended-spectrum β-lactamase
EUCAST	European Committee on Antimicrobial Susceptibility Testing
FN	Febrile neutropenia
G-CSF	Granulocyte colony-stimulating factor
IQR	Interquartile range
MDR	Multidrug-resistant
NSCLC	Non–small cell lung cancer
PD	Progressive disease
PDR	Pandrug-resistant
PR	Partial response
PS	Performance status
SD	Stable disease
SCLC	Small cell lung cancer
STs	Solid tumors
XDR	Extensively drug-resistant

## Appendix A

**Grade**	**ECOG PS**
0	Fully active; able to carry on all pre-disease activities without restriction.
1	Restricted in physically strenuous activity but ambulatory and able to carry out light work (e.g., light housework or office work).
2	Symptomatic; ambulatory and capable of all self-care but unable to carry out any work activities. Up and about more than 50% of waking hours.
3	Capable of only limited self-care; confined to bed or chair more than 50% of waking hours.
4	Completely disabled; cannot carry on any self-care; totally confined to bed or chair.
5	Death
